# Potentiometric Stripping Analysis of Cadmium and Lead with Constant Inverse Current in the Analytical Step Using an Open Tubular Mercury-Coated Glassy Carbon Electrode

**DOI:** 10.1155/2019/3579176

**Published:** 2019-04-02

**Authors:** Zvonimir J. Suturović, Snežana Ž. Kravić, Zorica S. Stojanović, Ana D. Đurović, Tanja Ž. Brezo-Borjan

**Affiliations:** Department of Applied and Engineering Chemistry, Faculty of Technology Novi Sad, University of Novi Sad, Novi Sad 21000, Serbia

## Abstract

The most important experimental parameters of the flow potentiometric stripping analysis (PSA) with oxygen as an oxidant were investigated and optimised. A simple, homemade flow system consisting of glassy carbon tubes, which served as a working and auxiliary electrode, was used. By applying a rest period before the stripping step (the flow stop mode) and by imposing a constant reductive current simultaneously with the interruption of potentiostatic control, significant increase of the flow PSA sensitivity was achieved. In the determination of cadmium and lead, quantitation limits of 0.11 and 0.82 *μ*g/L were obtained. The precision of the method was evaluated in terms of repeatability and reproducibility, with values of relative standard deviation lower than 4.0% for cadmium and 4.2% for lead. This modified technique was applied for simultaneous determination of cadmium and lead in milk, after a simple pretreatment of the samples by dilution and acidification. The method accuracy was confirmed by analysing the certified reference material of skimmed milk powder (ERM-BD151).

## 1. Introduction

The increasing need for automation of the electrochemical stripping analysis for its application in online measurements has resulted in the development of numerous flow systems, as well as specific techniques that enable sensitive, selective, and fast determination. In addition to the obvious benefit of online monitoring, flow techniques offer several important advantages over the stripping analyses performed in batch systems. The possibility of contamination of the sample is minimised, owing to minimum sample handling, that is, due to automation of the whole process. When replacing the solution before the stripping phase in order to improve selectivity and reduce interferences, errors occurring in batch systems can be easily avoided using the medium-exchange procedure. Exhaustion of the analyte during electrolysis is also avoided because of the constant flow of the solution, and finally, effective and reproductive mass transport is provided by flowing the solution. Furthermore, the application of flow injection analysis (FIA) [1–4] and sequential injection analysis (SIA) [5–7] enables faster, more precise, and versatile measurements and offers a unique possibility for automation.

Voltammetric stripping techniques are most often used in the context of flow measurements, primarily because of their high sensitivity. Various modifications of this technique, with a different character of initial signal in the analytical step, have been successfully used in these analyses since the beginning of the 70s of the last century [1, 8–13]. Potentiometric (PSA) [2, 14–19] and chronopotentiometric (ChSA) [17, 20–23] stripping analyses, which have somewhat less sensitivity than the voltammetric ones, due to the minor influence of the charging current on the stripping step, also have a wide range of applications in measurements in flow systems. Depending on the type of the sample, the required capacity and speed (frequency) of the measurement, numerous flow electrochemical cells have been applied. The most commonly used cell constructions are “flow through” [10, 12, 13, 21, 22, 24–28], “thin layer (channel),” [12, 15–20] and “wall-jet” [1, 29–31]. In recent times, graphite-felt electrode [32], carbon fibre-loaded electrode [5], microflow cell [33], microflow cell with screen-printed electrode [34, and screen-printed carbon nanotube electrode [6] have also been applied. Flow cells with tubular working electrode [8, 11, 35] are rarely used, despite the high capacity and easy maintenance, making them suitable for environmental monitoring.

The flow-stripping measurements are more often applied within the environmental surveillance, then within a food and beverages control, and in the clinical investigations, that is, the analysis of biological material. Numerous works deal with determining the trace metals (Zn, Cd, Pb, Cu, Hg, Ni, and Co) in various water samples, including wastewater [3, 10, 13, 21, 22, 27, 29], in food (heavy metals, As, and Se) [6, 12, 16, 18, 25, 26, 28], and in biological materials (Se) [20].

According to our knowledge, no research has been reported related to the application of flow PSA with a tubular working electrode, probably because of the low sensitivity of the method. This is particularly expressed in the analyses of non-deaerated solutions, when oxygen is used as an oxidant. However, this technique avoids the time-consuming deaeration step and prevents the sample contamination by the inert gas, as well as by chemicals that must be added as oxidants. Furthermore, because of its simplicity, flow PSA enables an easy automation of the whole process and its application in online measurement. The sensitivity of the determination by the PSA mode with dissolved oxygen as an oxidant can be improved significantly by application of constant inverse current in the analytical step [36]. The aim of this study was to increase the sensitivity of the flow PSA with a tubular working electrode under the above experimental conditions, by imposing a constant, reductive current during the analytical (stripping) step. The developed method was applied for the simultaneous determination of cadmium and lead in milk, after simple dilution and acidification of the sample.

## 2. Materials and Methods

### 2.1. Instrumentation

**Figure 1:**
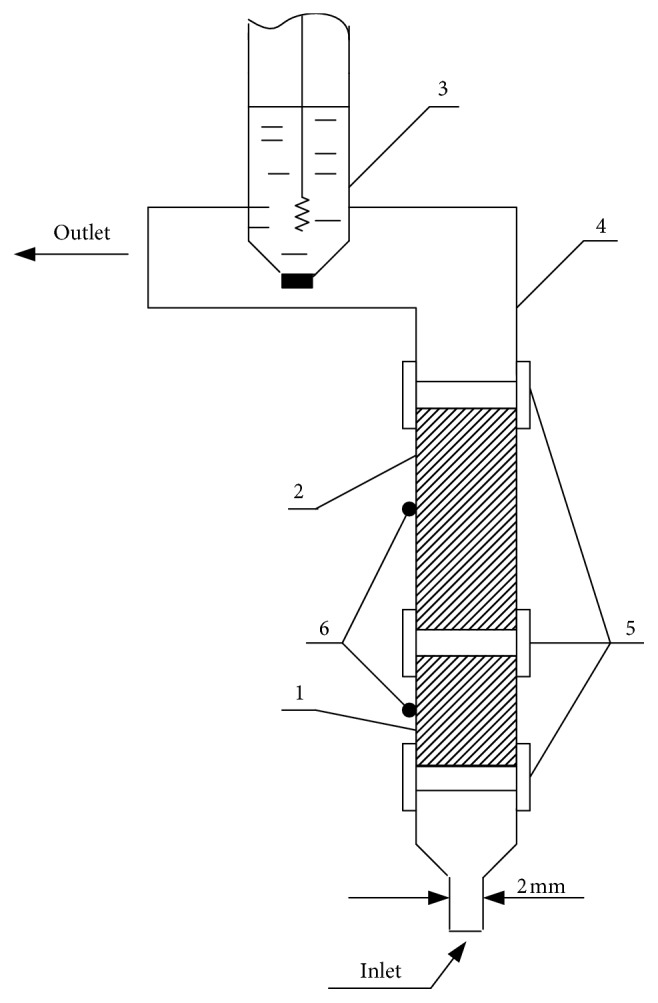
Open tubular flow cell. (1) Working electrode, glassy carbon tube (“Sigradur G”), *l* = 10 mm, *A*_in_ = 1.57 cm^2^. (2) Counter electrode, glassy carbon tube (“Sigradur G”), *l* = 50 mm. (3) Reference electrode, Ag/AgCl (3.5 mol/L KCl). (4) Reference electrode carrier. (5) Silicone hoses. (6) Electrical contacts.

All measurements were performed using an automatic system for potentiometric and chronopotentiometric stripping analyses of our own construction [36]. The homemade flow system (Figure 1) consisted of glassy carbon tubes connected by silicone hoses which served as working and counter electrodes, and of a usual Ag/AgCl (3.5 mol/L KCl) reference electrode. The solution flow was achieved by the multichannel system that consisted of a Marriotte's bottles, Teflon hoses (*d*_in_ = 2 mm), and magnetic valves controlled by the stripping analyser. The solution flow (*Q*) was maintained by the gravity flow, and flow rates were checked volumetrically in the waste.

### 2.2. Preparation of the Working Electrode

Mercury film electrode was formed by constant potential electrolysis (*E* = −0.90 V; formation time of 240 s; *Q* = 13.2 ml/min) from a separate solution containing 100 mg/L mercury(II) and 0.02 mol/L hydrochloric acid. Afterwards, the whole flow system was rinsed with triply distilled water for 240 s (*Q* = 13.2 ml/min). It was possible to carry out about 25 analyses of prepared samples at the same mercury film. After the analysis of each sample, the flow system was rinsed firstly with triple distilled water, then with 0.1 mol/L hydrochloric acid in ethanol (1 : 1), and with triply distilled water again. Each rinsing was performed during 240 s at the solution flow rate of 13.2 ml/min.

Because of the easy flow cell disassembly, the old mercury film was removed mechanically by a filter paper wetted firstly with acetone and then with triply distilled water. Alternatively, the mercury film renewal can be performed after the flow cell flushing with potassium dichromate (0.5 mol/L) during 240 s, in the solution flow of 13.2 ml/min, and then during 480 s with triply distilled water, at the same flow.

### 2.3. Reagents

Cadmium, lead, tin, indium, thallium, and mercury stock solutions (1 g/L) were prepared from high-purity SRM-3108, SRM-3128, SRM-3161, SRM-3214, SRM-3158, and SRM-3133 (NIST, USA), respectively, using triply distilled water. Hydrochloric acid was ultrapure grade (“Suprapur”, Merck), whereas nitric acid, ethanol, and potassium dichromate were analytical grade (Merck). All containers, vessels, and cells were washed with nitric acid (1 : 1), and then with distilled and triply distilled water.

### 2.4. Samples

The samples of pasteurised milk (five) with 2.8% milk fat (declared by producers) were randomly collected on the Novi Sad (Serbia) market. A skimmed milk powder-certified reference material (ERM-BD151) was used to evaluate the accuracy of the proposed method.

### 2.5. Pretreatment of the Samples

Prior to analysis, milk samples (20.00 g) were diluted with the triply distilled water (eightfold), acidified with hydrochloric acid to 0.08 mol/L, and then stirred intensively for five minutes.

Certified reference material was prepared by dissolution of 2.00 g of material with 100 ml of triply distilled water and then acidified with hydrochloric acid to 0.08 mol/L.

### 2.6. Statistical Analysis

The results were analysed by Student's *t*-test (*α* = 0.05) using OriginPro 8.0 software (OriginLab, USA).

## 3. Results and Discussion

### 3.1. Flow Stripping Potentiometry (Flow PSA)

Relatively low sensitivity of the PSA mode with the dissolved oxygen in the continuous flow analyses is caused by the fast transport of the oxidising agent which speeds up the deposit re-oxidation and so shortens the metal oxidation time (quantitative characteristic). In this work, the sensitivity of the applied PSA mode was considerably enhanced by stopping the solution flow and by applying a rest period before the stripping step, as well as by imposing a constant inverse current during the analytical (stripping) step. The rest period enabled an increase of the diffusion layer thickness and the electrochemical reduction of some oxygen in the retained solution as well. Both effects caused the slowdown of the metals' oxidation during the stripping phase, thereby increasing the oxidation time and the method's sensitivity. The imposition of the inverse (reductive) current simultaneously with the interruption of the potentiostatic control (at the end of the rest period) also enabled the metals' oxidation time to increase because of the reduction of some oxygen and partially because of the re-reduction of the freshly oxidised ions [36].

In all measurements, hydrochloric acid (0.08 mol/L) was used as an appropriate supporting electrolyte. Unlike the conditions of the sample pretreatment (for direct metals determination) in the previous work [36], in this investigation, more concentrated hydrochloric acid was used. Considering the adopted optimal deposition (electrolysis) potential for simultaneous determination of cadmium and lead of −1.15 V in the previous study [36], it was necessary to examine whether the hydrogen evolution has occurred on the working electrode at the applied experimental conditions. In order to compare the values of the cadmium and lead analytical signals (40 *μ*g/L of each metal), which were obtained by analysing the solutions acidified with 0.08 mol/L and 0.05 mol/L hydrochloric acid, five parallel analyses were performed (each analysis in three replications). A paired Student's *t*-test showed that the mean values of the obtained results do not significantly differ (*P*=0.95; *f* = 4; *t*_crit_ = 2.78; *t*_exp_ < *t*_crit_; *t*_Cd_ = 0.31; *t*_Pb_ = 0.23), which strongly indicates an absence of systematic errors; that is, eventually minor hydrogen evolution in more acidic medium does not affect electrolysis or stripping process, nor the stability of mercury film. Furthermore, the oxidation potentials of metals (qualitative characteristic) practically did not differ in the performed parallel analyses and were about −620 mV and −415 mV for cadmium and lead, respectively.

### 3.2. Influence of the Rest Period (Flow Stop)

As was mentioned, the sensitivity of the flow PSA with oxygen as an oxidant can be improved by the flow stopping prior to the stripping step. The only disadvantage of this PSA mode in relation to the mode without the flow breaking was the different mass transport during the electrolytic and stripping step, which disables a compensation of organic interferences in the direct analyses. Namely, in the conditions of the same mass transfer during the both PSA phases, the working electrode blockade by the surfactant compounds has the same influence on the electrolysis efficiency and the oxidant activity. Nevertheless, this problem can be overcome by applying the standard addition method.

**Figure 2:**
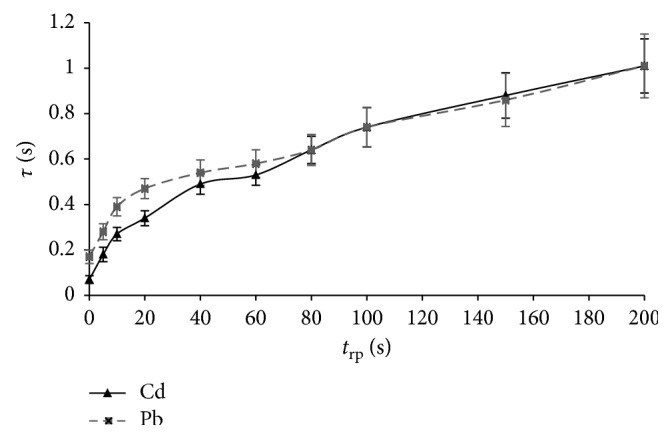
Influence of the rest period on the oxidation time (mean value ± 2 SD, *n*=5; *c*_m_ = 40 *μ*g/L; *t*_dep_ = 60 s; *Q* = 11.0 ml/min).

The influence of the rest period duration (*t*_rp_) after the flow stop on the oxidation time (*τ*) was explored in the range from 0 to 200 s, at a deposition time (*t*_dep_) of 60 s and a flow rate of 11.0 ml/min. This investigation was performed using different metal contents (*c*_m_ of 10, 40, and 80 *μ*g/L) in the supporting electrolyte. The results obtained by analysing the solution with the metal content of 40 *μ*g/L (mean value ± 2SD) are shown in Figure 2.

The first points on the obtained dependencies (“*τ*” intercepts) correspond to the results of the flow PSA without the flow break before stripping. The starting value of the lead analytical signal (*τ*) was somewhat larger than that of the cadmium one, considering that the supporting electrolyte and the value of the solution pH were more appropriate for the lead deposition during the electrolysis step. An increase in break time (rest period) up to about 30 s resulted in a significant increase of the metals' oxidation time. The character and response (relative sensitivity) of that part of dependencies for cadmium and lead were very similar. During this period, the method sensitivity increased primarily because of the increase of the diffusion layer thickness and partially because of the oxygen reduction. After stabilisation of the diffusion layer and amalgam homogenisation (*t*_rp_ ∼ 30 s), further increase of the oxidation time was only caused by the continuous oxygen reduction. Subsequently, by further increase of the rest period, the relative sensitivity (slope of dependence) gradually decreases for both metals, because of an ever-smaller amount of oxygen in the retained solution. In fact, for too long rest periods (*t*_rp_ > 200 s), the concentration of oxygen is approaching to some limit value, which depends on the volume of the flow cell and the potential of the electrolysis. The same character of the dependence of the analytical signal on the duration of rest period was obtained in the determination of cadmium and lead using a thin-layer cell [37, 38].

In the analyses of cadmium and lead solutions of lower concentrations, it was necessary to apply longer electrolysis or (and) longer rest periods. In order to perform sufficiently sensitive and reproductive analyses, the duration of pause must be in line with the analyte concentration and the deposition (electrolysis) time. Reproducibility of the analyses significantly decreased with too long rest periods (Figure 2) because oxidation of the deposit became more and more difficult because of the reduced content of oxygen. The rest periods in the range of 60–80 s were appropriate for the determination of metals' content of 40 *μ*g/L at the deposition time of 60 s. By applying a rest period of 200 s, after the metals' (10 *μ*g/L) deposition during the 200 s, relative sensitivity increased about 14 times for cadmium and about 6 times for lead in relation to PSA without flow break. The duration of the rest period did not affect the oxidation potential of metals.

### 3.3. Solution Flow Rate

The influence of the flow rate was examined in the range of 11–58 ml/min using solutions with the metals content in the range of 10–80 *μ*g/L, electrolysis time of 60 s, and a rest period of 60 s. Under the applied experimental conditions, the solution flow rate had no significant effect on the sensitivity of the metals determination. The used flow cell provided high electrolysis efficiency, whereas the appropriate sensitivity could be achieved at the flow of smaller volumes of the solution. The average discrepancies between the results obtained at different flow rates were 2.48% and 2.50% for cadmium and lead, respectively, which was in the range of the applied method reproducibility. This conclusion was additionally confirmed by analyses of the same volume of the solution, but at different deposition times and different flow rates, because the value of the analytical signal decreased linearly with the deposition time decrease, that is, the flow rate increase. Almost the same results were obtained by applying the thin-layer cell [37, 38]. The reproducibility of the analyses decreased at higher flow rates, probably because of the mechanical damage of the mercury film electrode. Considering the reproducibility of obtained analytical signals, the flow rate of 13.2 ml/min was selected as the appropriate one.

### 3.4. Deposition Time

**Figure 3:**
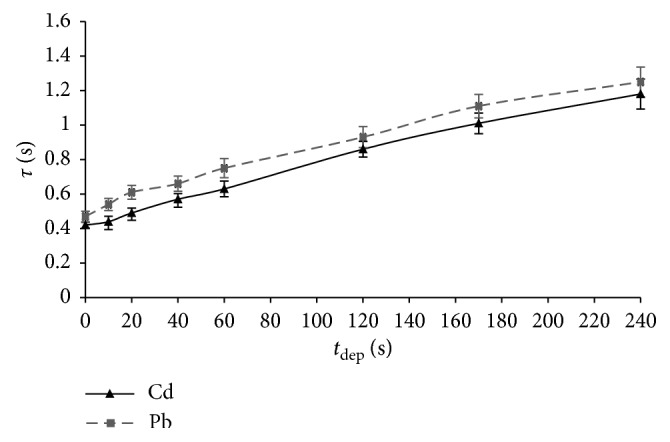
Dependence of the oxidation time on the deposition time (mean value ± 2 SD, *n*=5; *c*_m_ = 40 *μ*g/L; *t*_rp_ = 80 s; *Q* = 13.2 ml/min).

The effect of the deposition time, as an important experimental factor in PSA, was investigated in the range from 0 to 600 s at different rest periods.  Figure 3 shows the results of this investigation (mean value ± 2 SD, *n*=5) achieved by analysing the solution with the cadmium and lead content of 40 *μ*g/L, applying deposition times in the range of 0–240 s, the rest period of 80 s, and the flow rate of 13.2 ml/min.

The oxidation time increased linearly up to about 180 s, and then, a slight curvature of the dependence was occurred. This is typical for the stripping analysis when thin-layer electrodes are used and it is caused by the electrode saturation, which depends on the analyte concentration and electrolysis efficiency. The nonzero intercepts of dependencies were caused by the metal deposition during the rest period (80 s), but under the condition of diffusive mass transfer. Because of their relatively large values, it was possible to perform very reproductive analyses without the solvent flow, that is, without the electrolysis under the conditions of forced convection. The analysis of such a small volume of the retained solution in flow cell (0.2 ml) was possible due to the oxygen reduction during the rest period. The deposition time primarily depends on the analyte concentration, as well as on the rest period duration. An appropriate rest period can significantly reduce the required deposition time. For example, by applying the deposition time of only 60 s and rest period of 80 s, it was possible to determine cadmium and lead down to the content of 5 *μ*g/L. As was expected, the deposition time had no influence on the analyte oxidation potential.

### 3.5. Influence of the Reductive Constant Current

**Figure 4:**
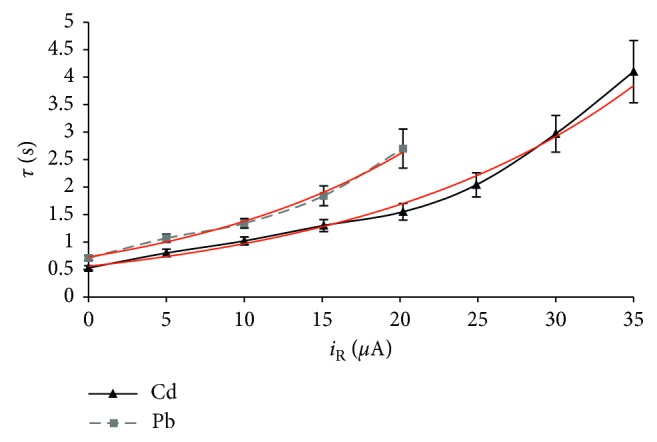
Influence of the reduction current on the oxidation time (mean value ± 2SD, *n*=5; *c*_m_ = 40 *μ*g/L; *Q* = 13.2 ml/min; *t*_dep_ = 120 s; *t*_rp_ = 60 s).

The reductive current must be carefully selected in order to enable only the slowdown of the metal oxidation. Namely, too large reductive current caused an electrode potential shift in a negative direction and break of oxidative PSA. In comparison with the results of previous research [36], in this work, it was possible to apply larger currents in a wider range, due to the larger surface of the working electrode (*A* = 1.57 cm^2^). The influence of the reduction current (*i*_R_) was examined in the range of 0–35 *μ*A, using the cadmium and lead solutions with the contents of 10, 40, and 80 *μ*g/L. The results obtained by analysing the solution with the metals' content of 40 *μ*g/L (mean values ± 2 SD, *n*=5), at the deposition time of 120 s, and rest period of 60 s, are shown in Figure 4. The oxidation time increased with the increase of the reduction current exponentially, for cadmium: *τ*=0.559 · *e*^0.055·*i*_R_^ (*R*^2^ = 0.9902), and for lead: *τ*=0.729 · *e*^0.064·*i*_R_^ (*R*^2^ = 0.9927).

For lower metal contents, it was necessary to apply larger currents in order to prevent fast oxidation of the small amount of deposit. However, the reproducibility of analysis decreased with the reductive current increase (Figure 4) due to the heavily stripping step, which is caused by complex processes of an additional oxygen reduction and re-reduction of the freshly oxidised metal ions. A larger sensitivity increase was possible to achieve for metals with more negative redox potential, because of the possibility of using larger reductive currents (Figure 4), which disable or terminate the oxidation of more precious metals, because of the electrode potential shift in the negative direction. In this case, the determination of less noble metals was facilitated by the use of more negative values of ending potentials. Furthermore, if the same value of the reductive current is applied, determination of less noble metal is much more reproductive (Figure 4). The nonzero intercepts of the shown dependencies (Figure 4) correspond to analytical signals obtained by the PSA with the flow stop. These values were smaller in comparison to the values of the oxidation times achieved by the flow-stop PSA at the same deposition time (120 s) (Figure 3), due to a shorter rest period that has been applied (60 s related to 80 s).

By using the reduction currents of 30.0 *µ*A and 15.1 *µ*A (*c*_m_ = 10 *μ*g/L; *Q* = 13.2 ml/min; *t*_dep_ = 200 s; *t*_rp_ = 60 s), an increase in method relative sensitivity of about 8 times for cadmium and about 4 times for lead, respectively, was related to flow PSA with the rest period use. It should be noted that these increases were achieved with a significant shorter rest period (60 s) in comparison to flow PSA without application of reductive current in analytical step (200 s, Section 3.2). The reductive currents, in the applied range, did not significantly affect the oxidation potentials of cadmium and lead. However, the application of too large reductive currents (near the critical value, which causes an electrode potential shift in a negative direction) caused shifting of the metals' oxidation potential to more negative values.

In Table 1 are shown the values of the relative sensitivity increase degree (*f*) of two modified PSA techniques, where PSA-*t*_rp_ is the flow PSA with flow break before the analytical step, while PSA-*t*_rp_-*i*_R_ represents the technique in which after the rest period, during the stripping phase, the reductive current is imposed. The *f*-values were calculated in comparison to the flow PSA without any modification (*t*_rp_ = 0, *i*_R_ = 0). The experimental conditions were the same (*c*_m_ = 10 *μ*g/L; *Q* = 13.2 ml/min; *t*_dep_ = 200 s), except the rest period which in the PSA-*t*_rp_ was 200 s, whereas in the PSA-*t*_rp_-*i*_R_ was only 60 s. As was mentioned, in the PSA-*t*_rp_-*i*_R_, the reductive currents of 30.0 *μ*A and 15.1 *μ*A were applied for the cadmium and lead determination, respectively.

### 3.6. Linearity of the Stripping Signal

The linearity of the elements' oxidation time was examined primarily to check the possibility of the standard addition method application for the calculation of the elements' content. The analyses were carried out in hydrochloric acid (0.08 mol/L); however, no analytical signals were detected, when deposition time of 360 s, rest period of 60 s, and reductive current of 15.1 *μ*A were applied. Considering the usual cadmium and lead contents in milk, linearity of the cadmium analytical signal was investigated in the range from 2 to 30 *μ*g/L (*t*_dep_ = 300 s; *t*_rp_ = 60 s; *i*_R_ = 15.1 *μ*A), whereas the content range for lead was from 40 to 100 *μ*g/L (*t*_dep_ = 120 s; *t*_rp_ = 60 s; *i*_R_ = 10.0 *μ*A). In these analyses, and also in the analyses of the real samples, a subtraction of the supporting electrolyte (or the sample matrix) potentiogram (base line) was applied. Correction of the sample potentiogram was performed by the analysis of blank or milk sample, by applying the deposition time (without the rest period) of only two seconds. The base line subtraction is necessary in the analysis of solutions with lower elements content, when the application of relatively large rest periods and reductive currents may cause an intensive stretch of the potentiogram.

**Figure 5:**
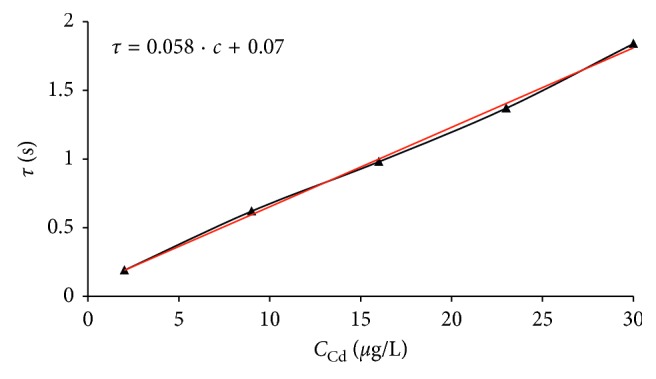
Linearity of cadmium analytical signal (*t*_dep_ = 300 s; *t*_rp_ = 60 s; *i*_R_ = 15.1 *μ*A; *Q* = 13.2 ml/min).

**Figure 6:**
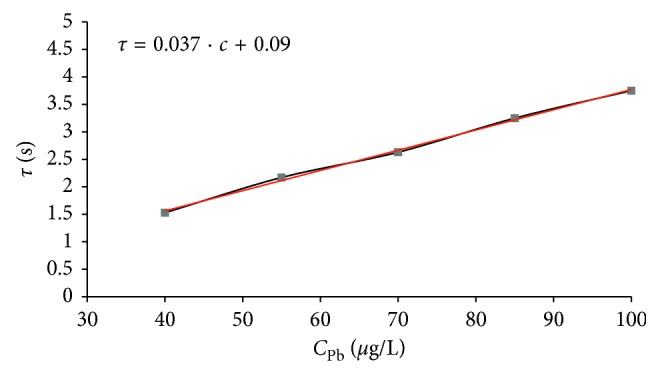
Linearity of lead analytical signal (*t*_dep_ = 120 s; *t*_rp_ = 60 s; *i*_R_ = 10.0 *μ*A; *Q* = 13.2 ml/min).

As a result of these experiments, very good linearity of the metals' analytical signal was obtained. Calibration in cadmium and lead concentration ranges yielded linear plots with average values (*n*=5) of slopes 0.058 and 0.037 s·L/*μ*g, intercepts of 0.07 and 0.09 s, and correlation coefficients of 0.996 and 0.991, respectively (Figures 5 and 6). The high values of the correlation coefficients, as well as a minor intercepts, confirmed the possibility of the standard addition method application. This opportunity has a great practical importance which was discussed in previous work [36]. However, out of investigated ranges, for the larger metals contents, a curvature of obtained dependencies was noticed (analogous to analyses at longer deposition times).

### 3.7. Quantitation Limit

The limit of quantitation (LOQ) was determined by the analyses of 0.08 mol/L hydrochloric acid (five replications). The deposition time of 660 s and rest period of 80 s were applied. The values of the reductive current were 32.6 *μ*A and 22.5 *μ*A for cadmium and lead, respectively. The quantitation limit was calculated according to the definition $\bar{X_{\text{b}}}+10\text{ SD}$ [39], where $\bar{X_{\text{b}}}$ was the mean value of the element contents in blank and SD (standard deviation). The limit of quantitation of 0.11 and 0.82 *μ*g/L for cadmium and lead was obtained, respectively. The reproducibility of these analyses, expressed as relative standard deviation (RSD), was for cadmium and lead, respectively, 6.7% and 5.1%.

The limits of quantitation for cadmium and lead obtained in previous work [36], where the PSA-*i*_R_ was applied in batch system, were 0.45 *μ*g/L (RSD 8.7%) and 2.3 *μ*g/L (RSD 5.9%), respectively (the reported detection limits were 0.30 and 1.7 *μ*g/L, respectively). In comparison to this method, flow PSA-*t*_rp_-*i*_R_ method proposed in this work provided more sensitive determination of cadmium and lead.

### 3.8. Precision

The precision of the method was evaluated in terms of the repeatability (expressed as intraday precision) and the reproducibility (intermediate precision). The repeatability of the method was investigated by the analyses of five independent solutions (three replications) containing 10 *μ*g/L of cadmium and lead and by applying the solution flow rate of 13.2 ml/min, deposition time of 200 s, rest period of 60 s, and reductive current of 15.1 *μ*A. In order to evaluate the method reproducibility, the described measurements were repeated in three consecutive days. The precision of the method was expressed as relative standard deviation. The repeatability of the method was 2.53% and 2.71% for cadmium and lead, respectively. The reproducibility of the method was 3.92% for cadmium and 4.19% for lead determination.

### 3.9. Interferences

The most common interferences in the electrochemical stripping analysis occur due to the formation of intermetallic compounds, overlapping of analytical signals, and the influence of organic compounds. These interferences were discussed in detail in previous paper [36], so only the interferences relevant to the determination of cadmium and lead in milk by flow PSA will be commented here. In connection with the interferences caused by the formation of the intermetallic compounds, no significant interferences of copper on the determination of cadmium were observed, due to the preplated mercury film which was used, as well as the standard addition method application. The overlapping interferences of thallium and indium on the cadmium analytical signal and tin on the analytical signal of lead were investigated in the matrix of blank (0.08 mol/L HCl) with addition of standard solution of cadmium (10 *μ*g/L) and lead (100 *μ*g/L). The criterion for interference was a relative error of less than ±5% within analytical determination of cadmium and lead. The results of this study are shown in Table 2. Because of the supporting electrolyte that was used, as well as the appropriate value of the deposition potential (−1.15 V), a good resolution of the metals signals, that is, the obvious inflection points of potentiogram, were obtained. No significant changes in quantitative and qualitative characteristics of determined metals were observed up to 30-fold greater contents of the investigated elements with similar oxidation potentials.

The obtained values of the relative errors (Table 2) were approximately the same related to values reported earlier [36], but were achieved at larger mass ratio of interfering ions to analyte (30 : 1 vs. 20 : 1). Improvement of selectivity has been achieved because of the high sensitivity of the developed method and the application of the base line subtraction. In the cases of larger mass ratios, the medium-exchange procedure must be applied, which is relatively simply in flow stripping techniques.

The presence of organic compounds (particularly surface-active) in the samples can complicate the stripping analysis. Surface-active substances tend to adsorb on the working electrode, thus inhibiting the elements deposition, as well as the stripping processes. Although in this study a direct analysis was applied, that is, the milk samples were prepared only by dilution and acidification, no difficulties were observed during the entire process of the flow PSA. Thanks to the significant dilution of samples (eightfold); the analytical signals were well defined and reproductive and the mercury film was very stable. However, the mercury film was less stable related to the previous investigation [36], in which the milk samples were prepared by microwave extraction, so the number of analyses that could have been done at the same mercury film was reduced from 30 to 25.

### 3.10. Method Accuracy

Accuracy of the defined method was tested by analysing the certified reference material of skimmed milk powder (ERM-BD151). The certified values of the cadmium and lead contents were 0.106 ± 0.013 and 0.207 ± 0.014 mg/kg, respectively. The uncertainty was expanded with a coverage factor *k* = 2 corresponding to the level of confidence of 95%. The experimental results, for cadmium (0.103 ± 0.015 mg/kg) and lead (0.203 ± 0.017 mg/kg), agreed well with the declared contents, considering that a Student's *t*-test showed that the mean values did not significantly differ (*P*=0.95, *f* = 4, *t*_crit_ = 2.78; *t*_exp_ < *t*_crit_; *t*_Cd_ = 0.892, *t*_Pb_ = 1.05). The determined metals contents were slightly lower than the certified values, which is expected, since the sample pretreatment did not include the destruction of organic matter. However, the results of the *t*-test indicate a high efficiency of the applied extraction, which was achieved because of the possibility of significant dilution of sample; in this way, interferences from organic substances were also reduced.

The validation parameters of the proposed method are summarized in Tables 3 and 4.

### 3.11. Analysis of Milk Samples

The five pasteurised milk samples were analysed after the simple dilution and acidification. Prepared extracts were analysed at a flow rate of 13.2 ml/min and deposition time of 300 s. Rest period and reductive current were 60 s and 20.2 *μ*A, respectively. The metals' contents were calculated by the method of two standard additions in order to obtain higher accuracy of the measurements, that is, the more dependable slope values. The obtained results (mean value ± 2 SD, *n*=5) are shown in Table 5.

The values of determined cadmium and lead contents were within the expected ranges [36]. According to the Serbian regulations [40], maximal permitted contents for cadmium and lead in milk are 0.01 mg/kg and 0.1 mg/kg, respectively. Taking the obtained results into consideration (Table 5), all the analysed samples were correct, although the lead content in one sample (M4) was near to highest permitted content. The possible cause of this can be contaminated raw material, the equipment used during the production process, packing materials, and storage conditions.

## 4. Conclusions

The flow PSA with the rest period before the stripping phase and with a constant reductive current during analyte oxidation enables direct, simultaneous determination of cadmium and lead in non-deaerated milk samples, after a simple pretreatment which includes only dilution and acidification. A rapid, sensitive, and selective method was developed, which is suitable for routine analyses, as well as for the monitoring of metals content (online analyses). Due to increase of the flow PSA sensitivity, a significant reduction of the analysis time was possible, as well as a larger sample dilution, which reduces organic interferences. Furthermore, the time-consuming deaeration of the sample is unnecessary, which simplifies the automation of the entire process and allows the application of the method in online analyses. Finally, all the analysed milk samples were correct, since the results of the real samples analysed by the proposed method were in accordance with the Serbian regulations regarding the maximum allowed metal content in milk.

## Figures and Tables

**Table 1:** The improvement of the flow PSA relative sensitivity.

Flow PSA mode	*f* ^a^	
	Cd	Pb
PSA-*t*_rp_	14	6
PSA-*t*_rp_-*i*_R_	112	24

^a^Values of the relative sensitivity increase degree.

**Table 2:** Interference study for cadmium and lead determination.

Metal	Interfering ion^a^	Change of signal (%)
Cd	Tl^+^	+2.20
	In^3+^	−3.95
Pb	Sn^2+^	+2.47

^a^The mass ratio of interfering ions to analyte was 30 : 1.

**Table 3:** Results of the analysis of the certified reference material of skimmed milk powder (ERM-BD151).

Element	Certified value (mg/kg)	Uncertainty^a^ (mg/kg)	Obtained value^b^ (mg/kg)	Bias (%)	*t*_exp_	*t*_crit_^c^
Cd	0.106	0.013	0.103 ± 0.015	−2.83	0.89	2.78
Pb	0.207	0.014	0.203 ± 0.017	−1.93	1.05	2.78

^a^As indicated in the ERM-BD151 certificate. ^b^Mean value ± 2 SD of five independent analysis of ERM-BD151. ^c^*P*=0.95; *f* = 4.

**Table 4:** The analytical characteristics of the proposed methods.

Parameter	Value	
	Cd	Pb
Limit of quantitation (*μ*g/L)	0.11	0.82
Repeatability (%)	2.53	2.71
Reproducibility (%)	3.92	4.19
Linearity		
Range (*μ*g/L)	2–30	40–100
Slope (s·L/*μ*g)	0.058	0.037
Standard deviation of the slope (s·L/*μ*g)	0.002	0.002
Intercept (s)	0.07	0.09
Standard deviation of the intercept (s)	0.003	0.002
Correlation coefficient	0.996	0.991

**Table 5:** Trace metals contents in milk samples.

Sample	Metal content (*μ*g/kg)	
	Cd	Pb
M^a^1	4.27 ± 0.39^*b*^	52.1 ± 4.6
M2	3.11 ± 0.34	65.3 ± 5.4
M3	3.85 ± 0.41	60.1 ± 5.1
M4	5.56 ± 0.59	96.8 ± 8.1
M5	5.27 ± 0.58	60.9 ± 4.9

^a^Milk. ^b^Mean value ± 2 SD, *n*=5.

## Data Availability

The data used to support the findings of this study are included within the article, and any further information is available from Dr. Zvonimir Suturović (zvonecos@tf.uns.as.rs) upon request.
